# Ensemble multi-retrieval methodologies with reasoning model decision in multi-step biomedical QA

**DOI:** 10.1093/database/baag061

**Published:** 2026-10-08

**Authors:** Jongmyung Jung, Hyeongsoon Hwang, Yein Park, Minju Song, Jaehoon Yun, Hyeon Hwang, Sanghoon Lee, Jiwoong Sohn, Jaewoo Kang

**Affiliations:** Department of Computer Science and Engineering, Korea University, 145 Anam-ro, Seongbuk-gu, Seoul, 02841, Republic of Korea; Department of Computer Science and Engineering, Korea University, 145 Anam-ro, Seongbuk-gu, Seoul, 02841, Republic of Korea; AIGEN Sciences, 58 Wangsimni-ro, Seongdong-gu, Seoul, 04778, Republic of Korea; Department of Computer Science and Engineering, Korea University, 145 Anam-ro, Seongbuk-gu, Seoul, 02841, Republic of Korea; AIGEN Sciences, 58 Wangsimni-ro, Seongdong-gu, Seoul, 04778, Republic of Korea; Department of Computer Science and Engineering, Korea University, 145 Anam-ro, Seongbuk-gu, Seoul, 02841, Republic of Korea; AIGEN Sciences, 58 Wangsimni-ro, Seongdong-gu, Seoul, 04778, Republic of Korea; College of Medicine, Hanyang University, 222 Wangsimni-ro, Seongdong-gu, Seoul, 04763, Republic of Korea; Department of Computer Science and Engineering, Korea University, 145 Anam-ro, Seongbuk-gu, Seoul, 02841, Republic of Korea; Department of Computer Science and Engineering, Korea University, 145 Anam-ro, Seongbuk-gu, Seoul, 02841, Republic of Korea; AIGEN Sciences, 58 Wangsimni-ro, Seongdong-gu, Seoul, 04778, Republic of Korea; Department of Biosystems Science and Engineering, ETH Zurich, Klingelbergstrasse 48, Basel, 4056, Switzerland; Department of Computer Science and Engineering, Korea University, 145 Anam-ro, Seongbuk-gu, Seoul, 02841, Republic of Korea; AIGEN Sciences, 58 Wangsimni-ro, Seongdong-gu, Seoul, 04778, Republic of Korea

## Abstract

Robust and trustworthy biomedical question answering (QA) remains a critical challenge for large language models (LLMs), especially in complex domains such as rare diseases, where information is fragmented across multiple sources. MedHopQA 2025 benchmark introduces 10 000 multi-step reasoning questions curated from Wikipedia, requiring systems to extract, connect, and synthesize biomedical knowledge across interlinked documents. In this work, we present a retrieval-augmented generation (RAG) and decision-making framework that integrates diverse retrieval strategies, including Query2Doc-based, Rationale-based, and Web-augmented retrieval, and employs a dedicated Decision Maker model to select or directly generate the most accurate and well-reasoned answers. Our system not only leverages evidence from both Wikipedia and the web but also explicitly evaluates and compares candidate answers to ensure answer reliability and reasoning transparency. Experiments on the test set demonstrate that our ensemble approach achieves state-of-the-art performance, yielding an Exact Match score of 0.84, and highlighting the importance of hybrid retrieval and robust decision-making in advancing biomedical multi-step QA. Furthermore, comprehensive analyses across retrieval stages reveal that mitigating model self-bias and ensuring a balanced integration of diverse sources are crucial for maximizing overall system performance, which ultimately reaches a score of 0.87.

## Introduction

Large language models (LLMs) have rapidly advanced the frontier of natural language processing (NLP), demonstrating remarkable capabilities across a range of domains. In biomedical and healthcare applications, however, the stakes are uniquely high: robust and trustworthy question answering (QA) systems are required to ensure reliable decision-making and knowledge dissemination. While numerous biomedical QA benchmarks, such as PubMedQA [1], BioASQ [2], and MedQA [3], have driven progress in single-hop factoid retrieval and reasoning, the ability of LLMs to perform multi-step reasoning remains insufficiently explored [4, 5], especially in complex domains like rare diseases, where information is dispersed and context-dependent [6, 7].

To address these limitations, the MedHopQA track at BioCreative IX [8] introduces a challenging benchmark: a collection of 10 000 expert-curated multi-step reasoning QA pairs derived from Wikipedia [9], focusing on diseases, genes, and chemicals, with an emphasis on rare conditions. Unlike prior datasets that typically target single-source fact extraction, MedHopQA questions require models to extract, connect, and synthesize information across multiple interlinked sources, mimicking the real-world process of clinical and biomedical inquiry.

In this work, we present a multi-step retrieval-augmented generation (RAG) and decision-making framework designed for MedHopQA. Our system combines multiple retrieval and reasoning strategies, integrating evidence from both Wikipedia and web. We leverage frameworks such as Query2Doc [10], Rationale-based retrieval, and Web search-augmented retrieval to generate candidate answers [11], and employ a dedicated Decision Maker model to select the most accurate and well-supported response, or to directly answer the question when candidates are insufficient. This ensemble approach ensures that both direct factual correctness and the underlying multi-hop reasoning are faithfully addressed.

Our method achieves state-of-the-art performance on the MedHopQA leaderboard, demonstrating the effectiveness of hybrid retrieval and robust LLM-based reasoning in tackling complex, multi-source biomedical QA. Beyond achieving top-tier results, we provide a comprehensive analysis of the underlying dynamics within our pipeline to illuminate the critical factors driving this performance. Crucially, our investigation reveals a pronounced phenomenon of model self-bias, where the Decision Maker exhibits a detrimental over-reliance on its own self-generated candidate answers, particularly those derived from web search augmentations. By demonstrating that a heterogeneous model configuration effectively mitigates this bias and enforces a balanced synthesis of diverse evidence, our findings offer valuable insights into the optimal design of robust, multi-agent ensemble architecture for complex biomedical reasoning. Furthermore, we evaluate the comparative efficacy of sparse versus dense retrieval and dissect the individual contributions of different LLMs across the generative retrieval and decision-making stages (Fig. 1).

**Figure 1:**
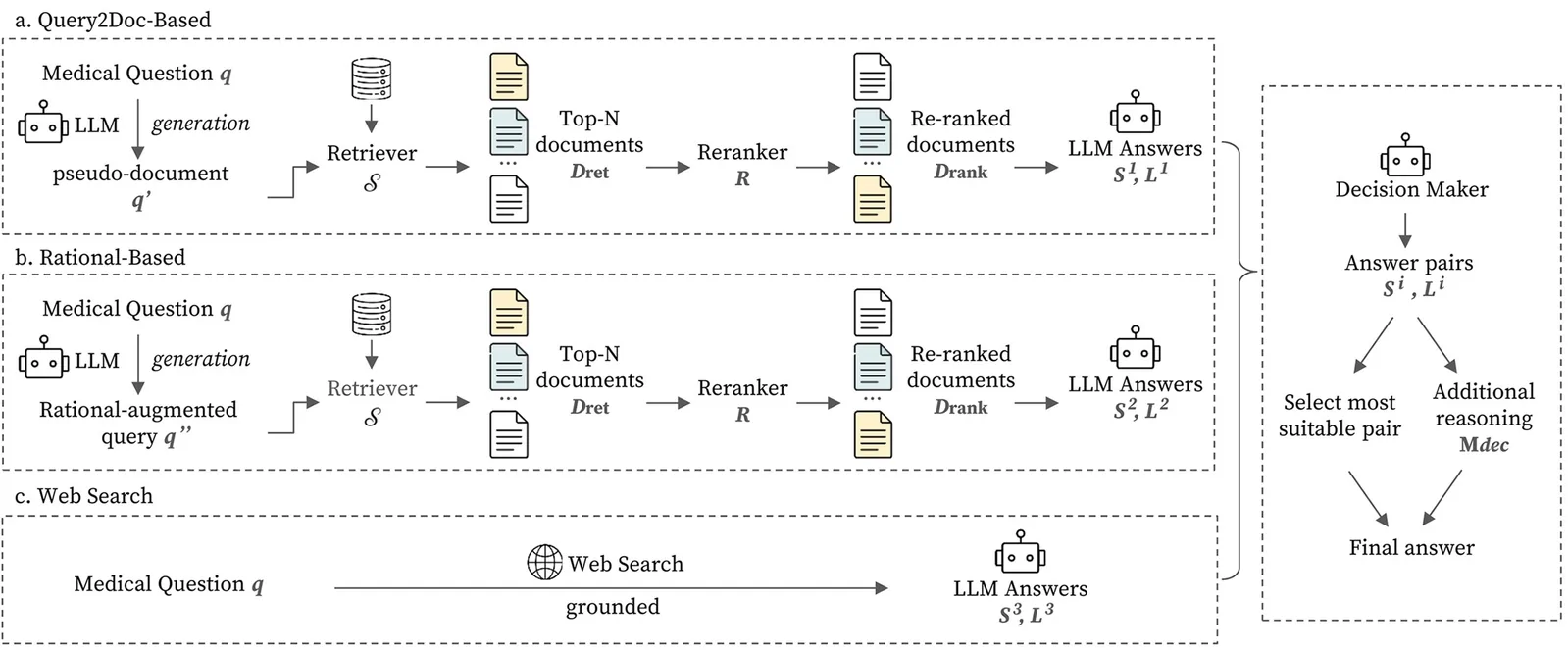
Overview of our multi-step RAG ensemble. Three answer candidates are generated via (a) Query2Doc-based with Wikipedia retrieval, (b) Rationale-based with Wikipedia retrieval, and (c) Web Search API applied. The Decision Maker then compares the candidates and selects the final answer pair or generates additional reasoning for the decision.

## Related work

### Multi-hop question answering benchmarks

Multi-hop QA has been widely studied as a way to evaluate the reasoning ability of QA systems over multiple documents. HotpotQA [12] provides a large-scale set of Wikipedia-based question–answer pairs that require reasoning across multiple supporting documents, along with sentence-level supporting facts that serve as supervision for explainable prediction. 2WikiMultiHopQA [13] combines unstructured Wikipedia text with structured Wikidata triples and annotates each question with an explicit reasoning path, addressing the observation that many questions in earlier datasets can be answered without genuine multi-hop reasoning. MuSiQue [14] composes single-hop questions in a bottom-up manner so that each reasoning step depends on a preceding one, yielding questions that are harder to solve through disconnected reasoning. These benchmarks, however, target the open domain and do not reflect the specialized and fragmented nature of biomedical knowledge. MedHopQA extends this line of work to the biomedical domain, focusing on diseases, genes, and chemicals with an emphasis on rare conditions, and requiring models to extract, connect, and synthesize information across multiple interlinked sources.

### Retrieval augmentation with LLM-generated queries

Addressing multi-hop questions requires gathering trustworthy evidence from multiple documents before a conclusion can be drawn [15–17], and retrieval-augmented LLMs have emerged as a powerful paradigm for this purpose in biomedical QA. This paradigm is directly relevant to the MedHopQA task, which requires multi-hop reasoning over various biomedical sources.

Recent works have increasingly focused on leveraging LLM-generated intermediate text (e.g. pseudo-documents, rationales) to guide retrieval towards relevant information. Query2doc [10] proposes a query-expansion strategy in which an instruction-tuned LLM is prompted to ‘write a passage that can answer the query’. The resulting pseudo-document is concatenated with the original question and submitted to a retriever. Because the synthetic passage typically introduces domain synonyms, acronyms, and MeSH terms absent from the user query, the method successfully recalls appropriate documents when searching biomedical repositories. Rationale-Guided Retrieval Augmented Generation (RAG²) [18] explicitly incorporates chain-of-thought reasoning into the retrieval loop. The LLM first breaks down the medical question by producing a multi-step rationale. Each rationale segment is treated as a refined search query, retrieving passages from diverse biomedical sources including PubMed, clinical guidelines, and textbooks, and a lightweight filter discards snippets that do not increase the rationale’s likelihood. The selected evidence is then fed back to the LLM to generate the final answer, thereby tightly coupling evidence collection with reasoning. These lines of work demonstrate complementary ways in which LLM-generated intermediate pseudo-documents, or explicit rationales, can guide retrieval towards information that supports multi-hop biomedical reasoning, a core requirement to MedHopQA.

### LLM ensemble and selection methods

Our framework builds on ensemble methods, which combine the outputs of multiple models to obtain more reliable predictions than any single model. This idea has recently been extended to LLMs, where candidate responses generated by different models are compared and selectively combined after generation. One line of work ranks the candidate outputs and fuses the top-ranked ones [19], while another adopts a selection-based approach that chooses the most suitable answer among the candidates, or abstains when none of them is reliable [20]. Our Decision Maker follows this selection-based ensemble paradigm: it compares the candidate answers produced by different retrieval strategies and selects the most suitable one, and when none of the candidates is deemed appropriate, it generates its own answer instead of abstaining, so that the pipeline always returns an answer.

## Preliminary study

To determine the optimal direction for our proposed methodology and identify the specific challenges inherent in the MedHopQA task, we conducted a preliminary study using the development set provided by the benchmark. This dataset consists of 45 multi-hop questions focused on healthcare and biomedical topics. Since the ground truth for these questions is explicitly constructed from information spanning two or more Wikipedia pages, we hypothesized that an RAG framework, grounded in Wikipedia, would significantly enhance answer accuracy compared to closed-book generation.

Based on this hypothesis, we prepared two distinct retrieval systems to compare their efficacy and efficiency: a dense retriever utilizing the publicly available Wikipedia embeddings from Cohere [21, 22], and a sparse retriever constructed by indexing a local Wikipedia dump via ElasticSearch [23, 24].

As summarized in Table 1, we first established a baseline by prompting GPT-4o [25] to generate answers without any external context, which yielded an Exact Match (EM) score of only 0.60. Incorporating the top 16 documents retrieved via the dense retriever significantly improved the EM score to 0.76. To address cases where the model failed to produce a definitive answer in this initial pass, we conducted a second trial with an expanded context of the top 32 documents for those specific unanswered questions. This multi-turn approach increased the final EM score to 0.82.

**Table 1.** Score comparison between various methods.^a^

Method	EM score
GPT-4o	0.60
GPT-4o + Dense Retrieval (single turn)	0.76
GPT-4o + Dense Retrieval (multi-turn)	0.82
GPT-4o + Sparse Retrieval (multi-turn)	0.80
*w/Query2Doc*	*0.87*

a Dense retrieval was conducted using vector embeddings generated by the Cohere Embed v3 model, leveraging the publicly available Cohere/wikipedia-2023-11-embed-multilingual-v3 dataset [22] hosted on Hugging Face. Sparse retrieval was conducted using an ElasticSearch built on a Wikipedia corpus. All methods except GPT-4o (first row) employed MedCPT as the reranker. Italicized value indicates the highest score achieved.

In parallel, we investigated a sparse retrieval strategy employing a multi-turn approach. Initially, we utilized the sparse retriever to provide context to GPT-4o for answer generation. To address queries that remained unresolved in this initial phase, we conducted a secondary pass using the o3 [26] model, which was prompted to answer directly without retrieved documents. This multi-turn strategy yielded an EM score of 0.80. Although this score was slightly lower than that of the dense retrieval setup, the sparse retriever offered significantly superior retrieval speed and operational efficiency, making it a compelling alternative for our framework.

As quantified in Table 2, the sparse approach reduced compute time by 33.6% and completely eliminated the heavy VRAM requirement (160.3 GB), operating efficiently within standard CPU memory instead.

**Table 2.** Comparison of compute time and memory usage for dense and sparse retrieval on the development set (45 questions).

Retrieval method	Time (s)	VRAM (GB)	CPU RAM (GB)
Dense	8.23	160.3	–
Sparse	5.47 (−33.6%)	–	33.2

Furthermore, we observed substantial improvements when queries were reformulated using the Query2Doc technique prior to document retrieval. We experimented with various numbers of retrieved documents to optimize the context window. Among these configurations, providing the top 200 documents yielded the highest performance, achieving an EM score of 0.87. This result underscores the significant benefit of query transformation in enhancing retrieval coverage and, consequently, answer accuracy.

However, a critical limitation observed during this preliminary phase was the inefficiency of the iterative process. As demonstrated by the multi-turn experiments, resolving complex queries often required an iterative workflow where failed attempts had to be identified and re-processed with different strategies. This reliance on manual intervention to manage such fallbacks posed a significant bottleneck. These findings highlighted the necessity for an automated pipeline capable of evaluating answer quality and dynamically selecting the most effective retrieval and reasoning strategy, thereby motivating the design of our proposed automated decision-making framework.

## Methods

To address the inefficiencies of the manual iterative process observed in our preliminary study, we designed a multi-step RAG and decision-making framework for the MedHopQA 2025 task, which involves generating both a short answer (the direct response) and a long answer (the supporting reasoning) for each question.

### Retrieval-augmented generation stage

Our RAG approach integrates multiple methodologies to robustly generate answers by leveraging various external knowledge sources from Wikipedia and the web. We detail our procedure as follows.

#### Query2Doc-based retrieval and answer generation

For a given question *q*, we first convert it to a search-oriented query $q^{\prime}$ using the Query2Doc approach. Query2Doc leverages an LLM to reformulate the original question into a more informative passage that can better assist in retrieving relevant information for answering the question:


\begin{eqnarray*}
q^{\prime}\ = \ \mathit{Query}2Doc\left( q \right)
\end{eqnarray*}

This query $q^{\prime}$ is then used to retrieve the top *N* number of documents ${{D}_{ret}}$ from a Wikipedia-based corpus using a retriever *S*:


\begin{eqnarray*}
{{D}_{ret}}\ = \ S\left( {q^{\prime},\ \mathit{Wikipedia}} \right)
\end{eqnarray*}

The retrieved documents are then passed through a reranker *R* to select the top *N* passages ${{D}_{{rank}}}$:


\begin{eqnarray*}
{{D}_{{rank}}}\ = \ R\left( {{{D}_{ret}}} \right)
\end{eqnarray*}

The refined document set ${{D}_{{rank}}}$ is provided as the context to model *M* to generate the short answer ${{s}^1}$ and the long answer ${{l}^1}$:


\begin{eqnarray*}
\left( {{{s}^1},\ {{l}^1}} \right)\ = \ M\left( {q,\ {{D}_{{rank}}}} \right)
\end{eqnarray*}

#### Rationale-based retrieval and answer generation

In parallel, we utilize a dedicated model ${{M}_{{rationale}}}$ to generate a rationale-augmented query. This approach uses an LLM to generate an answer containing rationale for the original question, which is then used as a query to retrieve documents relevant to that rationale-augmented perspective:


\begin{eqnarray*}
q^{\prime\prime}\ = \ {{M}_{{rationale}}}\left( q \right)
\end{eqnarray*}

This refined query $q^{\prime\prime}$ is then used to retrieve the top *N* number of documents from the Wikipedia corpus via the same retriever *S*, followed by passage selection through the reranker *R*:


\begin{eqnarray*}
{{D}_{{rank}}}\ = \ R\left( {S\left( {q^{\prime\prime},\ \mathit{Wikipedia}} \right)} \right)
\end{eqnarray*}

The final context ${{D}_{{rank}}}$ is given to the model *M* to generate both ${{s}^2}$ and ${{l}^2}$:


\begin{eqnarray*}
\left( {{{s}^2},\ {{l}^2}} \right)\ = \ M\left( {q,\ {{D}_{{rank}}}} \right)
\end{eqnarray*}

#### Web search-augmented retrieval and answer generation

To complement the above Wikipedia-based retrieval strategies, we employ a separate model ${{M}_{web}}$, which is specifically augmented with web search capabilities:


\begin{eqnarray*}
\left( {{{s}^3},\ {{l}^3}} \right)\ = \ {{M}_{web}}\left( q \right)
\end{eqnarray*}

### Decision-making stage

Throughout the decision-making process, we utilize a dedicated Decision Maker model ${{M}_{dec}}$. This model systematically compares the three candidate answer pairs generated by different retrieval-augmented methods, then selects the candidate it determines to be the most accurate and relevant, or directly answers the question itself if none of the candidates are deemed suitable. In this way, ${{M}_{dec}}$ ensures the generation of a reliable and accurate final response, even in cases where previous methods either fail to produce an answer or generate incorrect ones.

The three candidate answer pairs $\{ {( {{{S}^i},\ {{L}^i}} )} \}_{i = 1}^3$ generated from the retrieval-augmented methods (in the *Retrieval-augmented generation stage* under *Methods* section) are compared. The Decision Maker model ${{M}_{dec}}$ is employed to assess their accuracy, relevance, and internal consistency, thereby guiding to the most suitable answer pair.
If none of the three answers are deemed correct, an additional reasoning step is invoked. In this case, ${{M}_{dec}}$ directly answers the question *q* in question set *Q*.
  \begin{eqnarray*}
\left( {S,\ L} \right)\ = \ \left\{ {\begin{array}{@{}*{1}{l}@{}} {\left( {{{S}^i},\ {{L}^i}} \right)\ if\ a\ \mathit{suitable}\ \mathit{candidate}\ i\ \in \ \left\{ {1,\ 2,\ 3} \right\}\ \mathit{exists}\ }\\ {{{M}_{dec}}\left( Q \right)\ \mathit{otherwise}\ } \end{array}} \right.
\end{eqnarray*}

This ensures that final answer selection is consistently grounded in both external retrieval evidence and robust LLM-based reasoning.

## Experimental settings

### Dataset and evaluation metric

We evaluated our framework using the benchmark dataset of the MedHopQA track at BioCreative IX [8]. This dataset comprises 10 000 questions in the test set that require multi-step reasoning to answer correctly.

For performance assessment, we used the EM score. The official scorer of the BioCreative IX MedHopQA track judges each prediction by comparing it against the normalized lexical variations and accepted synonyms of the reference answer [8]. However, all EM scores reported in our analyses were computed by us using a standard Exact Match procedure (after lowercasing and article removal), rather than the organizers’ full scorer, because the organizers’ set of accepted lexical variations and synonyms is not publicly released. The official leaderboard figures are as reported by the organizers. Our reported EM is therefore a consistent and conservative lower bound on the official metric, ensuring that comparisons among our own experiments remain valid on a common basis.

### Model and document selection

Our methodology adopts a flexible RAG framework. While the underlying document retrieval and reranking mechanisms remained consistent across all experiments, we selected the most advanced models available at the time of Official Challenge phase and Post-Challenge phase to maximize performance. The complete list of utilized models along with their exact API identifiers and access dates is detailed in Table 9.

#### Document retrieval and reranking

For both phases, document retrieval is performed using ElasticSearch [23,24] built over a Wikipedia corpus. This corpus was constructed by chunking the raw text into segments containing a maximum of 100 words, with a 10-word overlap between adjacent chunks [27]. From this corpus, 1000 documents are initially retrieved. Subsequently, the MedCPT [28] cross-encoder is employed to rerank these candidates, selecting the top 200 documents to serve as the context for answer generation.

#### Model configuration

The specific model configurations for our final submission in the Official Challenge and the subsequent Post-Challenge phase are summarized in Table 3.

**Table 3.** Final submission scores across different model ensembles for the Official and Post-Challenge phases.^a^

	Stage				
Phase	Query2Doc	Rationale	Web Search	Decision Maker	EM score
Official	GPT-4o	o3, GPT-4o	Gemini 2.5 Pro	o3	0.84
Post-Challenge	Gemini 3 Pro	Gemini 3 Pro	Gemini 3 Pro	GPT-5.2	0.87

a In the Official Rationale setting, query transformation was performed by o3, with answer generation by GPT-4o.

**Official Challenge:** In our initial submission, we utilized GPT-4o for the Query2Doc generation and subsequent answer formulation. For the Rationale-based approach, we adopted o3 [26] for question reformulation to exploit its reasoning strengths, reverting to GPT-4o for the final answer generation. The Web Search component was powered by Gemini 2.5 Pro [29], and the final decision-making process was orchestrated by the o3 model. With the exception of the o3 model, which does not support temperature adjustment, all models were configured with a temperature of 0 to ensure deterministic outputs.

**Post-Challenge:** To further enhance performance, we transitioned to newer models. We employed Gemini 3 Pro [30] for all RAG stages, including Query2Doc, Rationale, and Web Search. For the final decision-making stage, we utilized GPT-5.2 [31]. Regarding hyperparameters, Gemini 3 Pro was configured with a temperature of 1.0, adhering to the guidelines provided in the official documentation. GPT-5.2 was configured with both ‘Reasoning Effort’ and ‘Verbosity’ set to ‘High’. These settings maximize internal reasoning computation and allow for more elaborated text generation, respectively.

## Results

We evaluated the efficacy of our framework by deploying distinct model combinations across two experimental phases. The performance outcomes for each phase are detailed in Table 3.

In the Official Challenge, our system achieved an EM score of 0.84, securing the first place among 13 competing teams. The comparative scores of the top five submissions on the leaderboard are presented in Table 4. Furthermore, in the Post-Challenge phase, the transition to our upgraded model configuration yielded a substantial performance improvement, elevating the EM score to 0.87. This result surpasses our official submission record, validating the effectiveness of integrating advanced models into our decision-making framework.

**Table 4.** Performance comparison of the top five teams on the official leaderboard. ^a^

Rank	Team	EM score
1	*DMIS Lab (ours)*	*0.84*
2	UETQuintet	0.83
3	Insilicom LLC	0.79
4	sparkle7160	0.73
5	PreceptorAI	0.73

a Italicized value denotes the highest score achieved.

## Analysis

For a more granular investigation into the efficacy of our framework, we carried out additional empirical analyses leveraging the test set of the MedHopQA 2025 benchmark.

### Performance analysis across stages

To thoroughly understand the performance dynamics of our pipeline, we evaluate the system at two distinct levels: the individual retrieval stages (Query2Doc, Rationale, and Web Search) and the final ensemble decision-making stage.

#### Retrieval-augmented generation stage

As detailed in Table 5, we established the scores from our official submission as baselines to measure the advancements brought by the latest models. For GPT-5.2, we investigated the impact of its specific hyperparameters, ‘Reasoning Effort’ and ‘Verbosity’, comparing its default configuration (None/Medium) against a maximized setting (High/High).

**Table 5.** Standalone performance comparison of different models across the three steps of the retrieval stage (Query2Doc, Rationale, and Web Search).^a^

Model	EM score
***Query2Doc***	
GPT-4o (Baseline)	0.78
GPT-5.2	
Reasoning Effort: None, Verbosity: Medium	0.78
Reasoning Effort: High, Verbosity: High	0.80
Gemini 3 Pro	*0.87*
***Rationale***	
o3 (Baseline)	0.82
GPT-5.2	
Reasoning Effort: None, Verbosity: Medium	0.78
Reasoning Effort: High, Verbosity: High	0.81
Gemini 3 Pro	*0.85*
***Web Search***	
Gemini 2.5 Pro (Baseline)	0.81
Gemini 3 Pro	*0.85*

a Italicized values indicate the highest score achieved within each step.

**Query2Doc:** In terms of performance, GPT-4o and the default configuration of GPT-5.2 yielded identical results. These were surpassed by the High/High configuration of GPT-5.2, with Gemini 3 Pro ultimately delivering the best performance in this stage.

**Rationale:** In this stage, o3 outperformed the default GPT-5.2. This performance gap suggests that o3, with its architectural specialization for reasoning, may be more naturally suited for rationale-based tasks than a general-purpose model operating under default settings. Although the High/High configuration of GPT-5.2 managed to close this gap, Gemini 3 Pro ultimately achieved the highest overall score in this stage.

**Web Search:** Gemini 3 Pro demonstrated a clear improvement over the baseline Gemini 2.5 Pro.

Across all individual retrieval stages, Gemini 3 Pro exhibited overwhelming superiority. For Query2Doc and Rationale—both of which rely on query transformation followed by sparse retrieval—Gemini 3 Pro’s exceptional performance suggests two key strengths. First, it is highly adept at enriching the transformed queries with precise medical terminology, synonyms, and relevant entities, which directly enhances the efficacy of the sparse retriever. Second, it demonstrates a superior ability to accurately infer and extract the correct answer from the retrieved documents. This robust comprehension extends seamlessly to the Web Search stage, where the model effectively filters and captures core information from the diverse and broad contexts retrieved from the open web.

#### Decision-making stage

To isolate the impact of the Decision Maker, we conducted experiments where the models for the Query2Doc and Rationale stages were fixed to either GPT-5.2 (High/High) or Gemini 3 Pro, while the Decision Maker model was varied. The results are summarized in Table 6.

**Table 6.** Performance comparison of different Decision Maker models with fixed retrieval inputs.^a^

Query2Doc	Rationale	Web Search	Decision Maker	EM score
GPT-5.2	GPT-5.2	Gemini 3 Pro	o3	0.86
			GPT-5.2	0.86
			Gemini 3 Pro	0.85
Gemini 3 Pro	Gemini 3 Pro	Gemini 3 Pro	GPT-5.2	*0.87*
			Gemini 3 Pro	0.84

a Note that GPT-5.2 was utilized with both ‘Reasoning Effort’ and ‘Verbosity’ set to the ‘High’ configuration. Italicized value indicates the highest score achieved.

When the generative retrieval models were fixed to GPT-5.2, utilizing either o3 or GPT-5.2 as the Decision Maker yielded an identical EM score of 0.86. However, substituting Gemini 3 Pro into the decision-making role resulted in a slight performance decline to 0.85.

This contrast became even more pronounced when the generative models were fixed to Gemini 3 Pro. In this configuration, deploying GPT-5.2 as the Decision Maker achieved the highest overall score of 0.87. Conversely, when Gemini 3 Pro was utilized as the Decision Maker for its own generated candidates, the performance dropped substantially to 0.84.

These findings reveal a compelling dichotomy: despite Gemini 3 Pro’s exceptional and leading performance in individual generative retrieval tasks, its role as an evaluator and Decision Maker is relatively suboptimal. The contextual background and underlying causes of this phenomenon will be further investigated in the subsequent section detailing the Decision Maker’s preference analysis.

#### Beyond numerical parity: reasoning depth and explainability

A notable observation from our stage-wise results is that the standalone Query2Doc configuration (Gemini 3 Pro) achieves an EM score of 0.87 (Table 5), identical to our full ensemble pipeline (Table 6). This numerical parity, however, does not imply equivalence in reasoning capability. Instead, it demonstrates that while a multi-stage ensemble with adjudication does not always surpass the best individual component on EM metrics—particularly when a single model performs at a high ceiling—its primary architectural value lies in substantially enhancing explainability, rationale precision, and decision transparency. Because EM evaluates strictly whether a normalized short answer matches the ground truth—which often reduces to a single biomedical entity or a binary response—it inherently masks the soundness, specificity, and transparency of the underlying reasoning paths. The distinct contribution of the decision-making stage manifests instead in the quality of the generated long answer, specifically the supporting rationale and the auditability of the final selection.

We characterize this qualitative value along two primary axes, detailed through representative case studies in Supplementary Appendix D. The first axis is reasoning depth: by explicitly evaluating candidate answers against rigorous criteria such as accuracy and entity precision, the Decision Maker selects or refines a response that is better grounded in evidence than any single-step output. This is illustrated in our first case study, where both configurations arrive at the same short answer, but the full pipeline promotes a far more precise and well-supported rationale—representing a substantial architectural advantage invisible to the EM metric. The second axis is explainability: because the final selection involves an overt adjudication among multiple candidates, it provides a documented rationale detailing why the chosen answer was preferred. As shown in our second case study, this comparative mechanism can also deviate from the standalone output to correct a plausible but insufficiently precise biomedical entity, thereby enhancing both clinical explainability and quantitative correctness.

Together, these cases demonstrate that our decision-making framework operates through a single, consistent mechanism of criteria-based comparison. This approach enhances accuracy in general scenarios while preserving a distinct advantage in reasoning depth and explainability even where macro-metrics saturate.

### Comparative analysis of sparse and dense retrieval

In our preliminary study, we utilized publicly available Wikipedia embeddings provided by Cohere to construct the dense retrieval system. However, these embeddings were generated from a November 2023 snapshot. To mitigate temporal misalignment and better reflect the information landscape during the Official Challenge, we constructed a new dense index for this study using the Qwen3-embedding-8b [32] model, extracting embeddings from a November 2025 Wikipedia dump [33]. This system was coupled with Qwen3-reranker-8b [32] for the reranking stage. To fully exploit the extended context window supported by the Qwen3 reranker—which exceeds that of MedCPT—we adjusted our chunking strategy for the dense index. Text was segmented into 800-word chunks with a 160-word overlap to maximize contextual continuity, a distinct configuration from the shorter segments employed in our ElasticSearch-based sparse retrieval.

The comparative results are summarized in Table 7. When transitioning from sparse to dense retrieval with GPT-5.2, we observed a marginal improvement in the Query2Doc stage, where the score rose from 0.80 to 0.82. However, the Rationale-based stage showed no variation, maintaining a score of 0.81 across both retrieval methods. Conversely, with Gemini 3 Pro, the trend was mixed: Query2Doc performance actually declined from 0.87 to 0.85 when using dense retrieval, while the Rationale stage exhibited a slight increase from 0.85 to 0.86.

**Table 7.** Performance comparison between Sparse and Dense retrieval methods across the Query2Doc and Rationale steps.^a^

	EM score	
Model	Query2Doc	Rationale
***GPT-5.2***		
w/Sparse Retriever	0.80	0.81
w/Dense Retriever	0.82	0.81
***Gemini 3 Pro***		
w/Sparse Retriever	*0.87*	0.85
w/Dense Retriever	0.85	*0.86*

a Two distinct models were evaluated with each retriever type to assess the impact of retrieval strategy. Italicized values denote the highest score achieved within each step.

In terms of speed and efficiency, the sparse retriever demonstrated a clear advantage over dense retrieval, echoing the preliminary findings on the dev set. As shown in Table 8, it achieved a substantial 42.5% reduction in compute time on the full test set of 10 000 questions, while completely bypassing the heavy 140 GB VRAM requirement by operating within 36.5 GB of CPU RAM.

**Table 8.** Comparison of compute time and memory usage for Dense and Sparse Retrieval on the test set (10 000 questions).

Retrieval method	Time (s)	VRAM (GB)	CPU RAM (GB)
Dense	2214.29	140	−
Sparse	1275.16 (–42.5%)	−	36.5

**Table 9.** API identifiers and access dates of the language models utilized in the official runs and post-challenge evaluations.

Model	API identifier	Access date
***Official***		
o3	o3-2025-04-16	1 June 2025
GPT-4o	gpt-4o-2024-08-06	
Gemini 2.5 Pro	gemini-2.5-pro-preview-05-06	
***Post-challenge***		
o3	o3-2025-04-16	1 March 2026
GPT-5.2	gpt-5.2-2025-12-11	
Gemini 3 Pro	gemini-3-pro-preview	

Overall, given that the performance fluctuations between the two retrieval methods were minimal, the clear advantages in processing speed and hardware requirements make the sparse retrieval approach adopted in our Official and Post-Challenge settings the optimal choice for this task, offering superior operational efficiency without compromising accuracy.

### Analysis of Decision Maker's direct-generation fallback mechanism

A key architectural strength of our proposed framework is the direct-generation fallback mechanism embedded within the Decision Maker. Standard selection-based routing or ensemble methods are inherently upper-bounded by the quality of the candidate pool. If all generation pathways converge on a false consensus due to shared retrieval blind spots or superficial text matching, a pure ranking or selection model is forced to propagate a suboptimal answer.

To mitigate this limitation and achieve architectural completeness, our Decision Maker is designed to critically evaluate the collective sufficiency of the candidate pool. When all candidates are deemed inadequate to address the specific nuances of the query, the Decision Maker bypasses the selection process entirely and independently formulates a response using its internal parametric knowledge.

In our experiments, this fallback mechanism served as an essential safety net. Under our best-performing configuration—employing Gemini 3 Pro for the candidate generators (Query2Doc, Rationale, and Web Search) and GPT-5.2 as the Decision Maker—the model resorted to direct generation in 2.7% of the total cases. This empirical result demonstrates that while retrieval-stage consensus is generally reliable, a non-negligible portion of complex queries requires a higher-level critical adjudication that transcends the provided context. For a comprehensive qualitative analysis and specific case studies demonstrating this capability, please refer to Supplementary Appendix E.

### Analysis of Decision Maker preferences

To understand the decision-making dynamics within our proposed pipeline, we analysed which retrieval strategy the Decision Maker prioritized when formulating the final answer. We quantified this preference by calculating the proportion of instances where the Decision Maker’s final answer matched the output of a single retrieval step exclusively.

#### Preference trends and self-bias

The results, visualized in Fig. 2 with detailed scores in Table 6, reveal a consistent trend across all experimental combinations: the Decision Maker exhibited a marked preference for the Web Search step. Notably, when Gemini 3 Pro was employed as the Decision Maker, this preference for the Web Search step was exceptionally high. Prior work has shown that LLM evaluators tend to recognize and favour their own generations when used as judges [34]. Consistent with these findings, our results indicate that the Decision Maker exhibits a similar self-bias: its disproportionate reliance on the Web Search candidates stems from the fact that those candidates were generated by Gemini 3 Pro itself.

**Figure 2:**
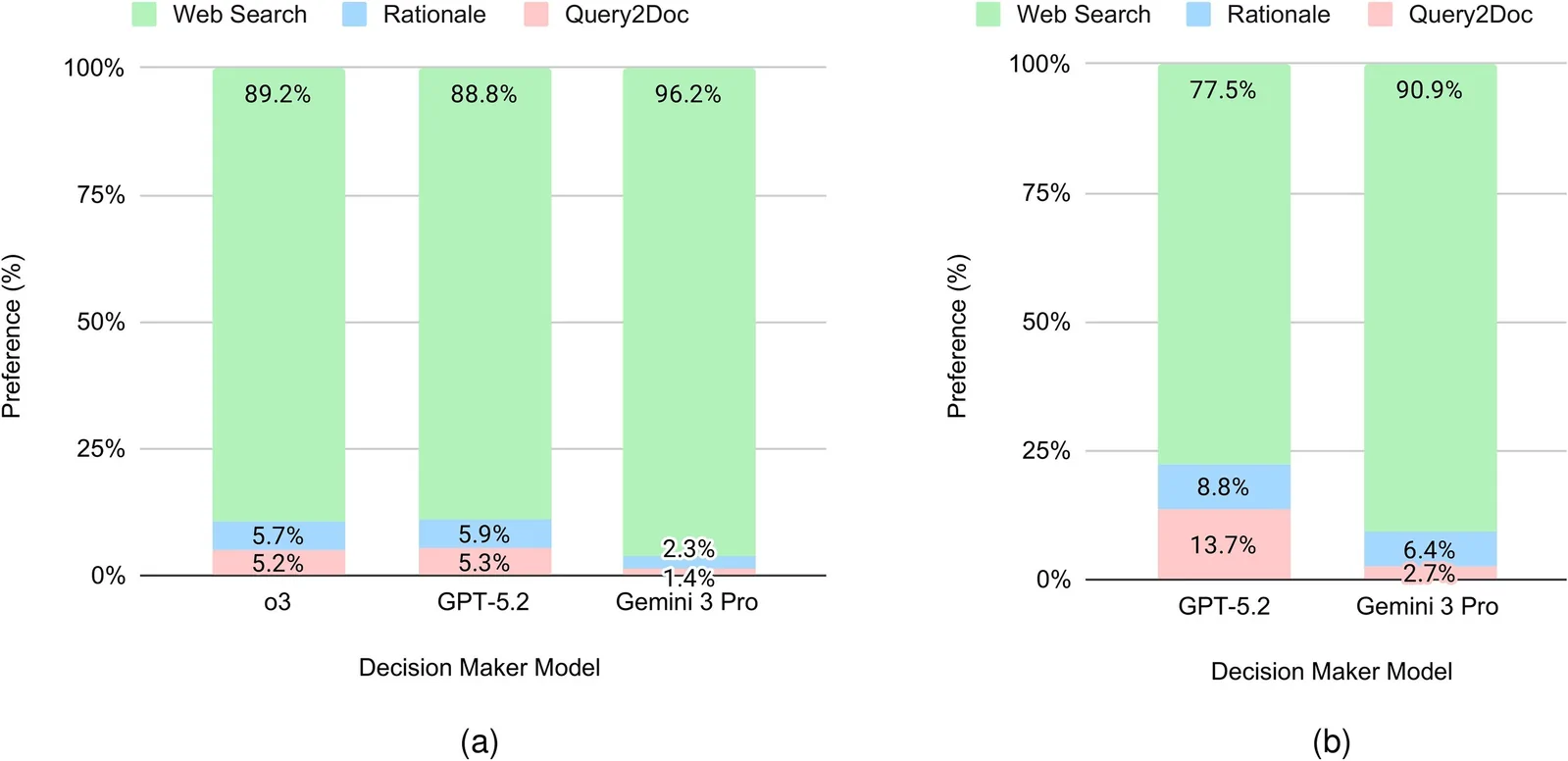
Analysis of source preferences across different Decision Maker models. Model configuration for RAG stage: (a) Query2Doc and Rationale steps by GPT-5.2, Web Search by Gemini 3 Pro, (b) all RAG steps by Gemini 3 Pro.

#### Correlation with performance

Crucially, we observed a negative correlation between excessive reliance on the Web Search step and overall performance. The data suggest that performance degrades when the Decision Maker becomes over-dependent on a single retrieval source.

This hypothesis is strongly supported by the Gemini 3 Pro (Query2Doc/Rationale/Web Search) + GPT-5.2 (Decision-Making) combination. While the Decision Maker in this configuration also favoured the Web Search step, it demonstrated a significantly lower dependency on this source compared to other experimental configurations. By mitigating the tendency to rely heavily on a single dominant step and maintaining a more balanced evaluation of diverse candidate answers, the model successfully synthesized information from across the pipeline. This capacity to consider alternative possibilities beyond the Web Search results was a key factor in achieving the highest overall performance of 0.87.

## Conclusion

In this study, we presented a comprehensive framework designed to address the challenges of multi-hop biomedical reasoning. We propose a RAG and decision-making framework that leverages diverse retrieval strategies, including Query2Doc-based, Rationale-based, and Web-augmented retrieval, coupled with a robust Decision Maker model to select or generate the most accurate and well-reasoned answers.

Our results demonstrate that combining multiple retrieval and reasoning frameworks can significantly enhance the ability of LLM-based systems to handle complex, multi-hop biomedical questions, particularly in domains such as rare diseases where knowledge is both specialized and interconnected. Our approach not only secured the top position on the official leaderboard but also achieved further performance gains in the post-challenge phase, confirming the robustness and effectiveness of ensemble retrieval and decision-based answer selection.

## Limitation

**Dependency on proprietary models**. Our framework relies on commercial closed-source APIs. Because these models are frequently updated behind closed doors, unexpected shifts in their internal behaviour pose challenges to long-term reproducibility.

**Latency and financial cost**. The pipeline’s sequential architecture—requiring multiple API calls for Query2Doc, Rationale generation, and final decision-making—accumulates notable network latency and financial overhead. This limits immediate scalability for real-time or large-scale deployments.

**Web search dynamics and contamination**. While live web search enhances retrieval coverage, the dynamic nature of web data inherently compromises result consistency over time. Moreover, post-challenge web indexing increases the risk of data contamination, such as retrieving Wikipedia mirrors or explicit public solution write-ups.

## Supplementary Material

baag061_Supplemental_File

## Data Availability

The data underlying this article were derived from publicly available resources described in the manuscript. The MedHopQA benchmark dataset is available through the BioCreative IX challenge at https://www.ncbi.nlm.nih.gov/research/bionlp/medhopqa. The code used to conduct the experiments in this study is available on GitHub at https://github.com/dmis-lab/bc9-medhop-qa.
