# Supplementary material for: Ensemble multi-retrieval methodologies with reasoning model decision in multi-step biomedical QA
Source: Database (Oxford). 2026 Oct 8;2026:baag061. doi: 10.1093/database/baag061 (PMC13648003; doi:10.1093/database/baag061)
Supplement: Supplementary file 1 [file baag061_supplemental_file.doc]

## **Appendix A**

**Model Specifications and Versioning**

For reproducibility, we report the api identifier used in each phase and the date of access.

[Table 9 goes here]

## **Appendix B**

**Prompts**

This section presents the exact prompts utilized during our experiments.

**Prompts to generate answer**

**System Prompt**

You are a medical QA assistant.

The following is a question about medical knowledge.

You are given multiple retrieved documents as reference context.

1. Use **only** the information contained in those documents.

- Do **NOT** hallucinate or invent facts.

2. Think step-by-step:

• First, reason through the relevant information.

• Then give a **concise Short Answer** (1-3 words).

3. Short Answer formatting rules

• Preserve the entity's **full official name**.

- Type 2 → “Diabetes mellitus, type 2

- Factor VIII → Factor VIII deficiency

- 2 → “Chromosome 2

- Carpenter's → Carpenter's syndrome

- NPH → Normal Pressure Hydrocephalus

• For yes/no questions, reply **exactly** Yes or No.

4. You are strongly required to follow the specified output format;

conclude your response with the phrase "Therefore the answer is [ANSWER].".

**User Prompt**

{question or pseudo-document}

Retrieved context:

[1] Title: {title_1}

Text: {text_1}

[2] Title: {title_2}

Text: {text_2}

...

[N] Title: {title_N}

Text: {text_N}

**Prompts of Query2Doc-based query reformulation**

**System Prompt**

You are a medical QA assistant.

The following is a Write a medical passage that can help answer the given query.

Include key information or terminology for the answer.

Example:

Query: A 39-year-old woman presents to the family medicine clinic to be evaluated by her physician for weight gain. ... Which of the following recommendations may be appropriate for the patient at this time? A) Hepatitis B vaccination B) Low-dose chest CT C) Hepatitis C vaccination D) Shingles vaccination

Passage: Against vaccine-preventable diseases. Every visit by an adult to a healthcare provider should be an opportunity to provide this protection. ...

Query: A 23-year-old male presents to his primary care physician after an injury during a rugby game. ... Which of the following is the most likely diagnosis? A) Medial collateral ligament tear B) Lateral collateral ligament tear C) Anterior cruciate ligament tear D) Posterior cruciate ligament tear

Passage: Diagnosing PCL Injuries: History, Physical Examination, Imaging Studies, Arthroscopic Evaluation. ...

Query: A 45-year-old woman is in a high-speed motor vehicle accident and suffers multiple injuries ... Which of the following is an appropriate next step? A) Provide transfusions as needed B) Withhold transfusion based on husband's request C) Obtain an ethics consult D) Obtain a court order for transfusion

Passage: Legal and ethical issues in safe blood transfusion. ...

Query: A 4-year-old male is accompanied by his mother to the pediatrician. ... Which of the following would most likely be found on biopsy of this patient's kidney? A) Mononuclear and eosinophilic infiltrate B) Replacement of renal parenchyma with foamy histiocytes C) Destruction of the proximal tubule and medullary thick ascending limb D) Tubular colloid casts with diffuse lymphoplasmacytic infiltrate

Passage: The natural history of urinary infection in adults. ...

**User Prompt**

Query: {question}

Passage:

**Prompts of Rationale-based query reformulation**

**System Prompt**

You are a medical expert.

**User Prompt**

{question}

**Prompts of Web Search-augmented retrieval stage**

**System Prompt**

You are a medical QA assistant.

The following is a question about medical knowledge.

You will use **Google Search** to retrieve relevant information. This retrieved information will serve as your **sole reference context**.

1. Base your answer **exclusively** on the information retrieved through your Google Search.

*	Do **NOT** use any prior knowledge.

*	Do **NOT** hallucinate or invent facts.

2. Think step-by-step:

*	First, identify the key information from your search results relevant to the question.

*	Then, reason through this retrieved information to formulate your answer.

*	Finally, provide a **concise Short Answer** (1-3 words).

3. Short Answer formatting rules:

*	Preserve the entity's **full official name**.

*	Type 2 → “Diabetes mellitus, type 2”

*	Factor VIII → “Factor VIII deficiency”

*	2 → “Chromosome 2”

*	Carpenter's → “Carpenter's syndrome”

*	NPH → “Normal Pressure Hydrocephalus”

*	For yes/no questions, reply **exactly** "Yes" or "No".

4. You are strongly required to follow the specified output format; conclude your response with the phrase "Therefore the answer is [ANSWER].".

**User Prompt**

{question}

**Prompts of decision-making stage**

**System Prompt**

You are an expert decision-making assistant.

Please carefully analyze these three answers and choose the **best** one. Follow these steps:

1. **Identify the key decision criteria** (e.g., accuracy, relevance to the question, logical consistency, completeness).

2. **Compare each candidate answer** step-by-step with respect to these criteria. Clearly show your reasoning process.

3. **Decide whether any of the three answers is appropriate and accurate enough to be selected.**

- If **one** candidate is suitable, select it and explain why it is better than the other two.

- If **none** of the candidates are suitable, provide your own short and long answers that directly address the question, and explain why the candidates were not appropriate.

4. **Output the final decision** as the final selected long answer or your own generated long answer.

When you output an answer, please follow these rules:

- Show your full reasoning process as a step-by-step explanation before providing the final decision.

- Short Answer formatting rules

• Preserve the entity's **full official name**.

- Type 2 → “Diabetes mellitus, type 2

- Factor VIII → Factor VIII deficiency

- 2 → “Chromosome 2

- Carpenter's → Carpenter's syndrome

- NPH → Normal Pressure Hydrocephalus

• For yes/no questions, reply **exactly** Yes or No.

- If you generate your own answers, do so with the same thoroughness and format.

- You are strongly required to follow the specified output format; conclude your response with the phrase "Therefore the answer is [ANSWER]."

**User Prompt**

Here is the original question:

{question}

Below are three candidate answers, each with a short and long version:

1. Candidate A:

- Short Answer: {Short answer from Web Search-augmented retrieval}

- Long Answer: {Long answer from Web Search-augmented retrieval}

2. Candidate B:

- Short Answer: {Short answer from Rationale-based retrieval}

- Long Answer: {Long answer from Rationale-based retrieval}

3. Candidate C:

- Short Answer: {Short answer from Query2doc-based retrieval}

- Long Answer: {Long answer from Query2doc-based retrieval}

## **Appendix C**

**Qualitative Examples of Model Responses from Each Pipeline Stage**

This section presents the actual responses generated by the models at each stage of our proposed framework. For the examples provided below, Gemini 3 Pro was utilized for the RAG stage, while GPT-5.2 was employed for the decision-making stage.

**Case of the Decision Maker Selecting from Candidate Answers**

**Question**

Parsonage-Turner syndrome and hereditary neuralgic amyotrophy (HNA) are distinct conditions that both involve nerve damage and muscle atrophy related to brachial plexus degeneration. Which thick triangular muscle is the most likely to be affected by both conditions?

**Query2Doc**

**Short Answer**

Deltoid muscle

**Long Answer**

Based on the retrieved documents, **Hereditary neuralgic amyotrophy (HNA)** and **Parsonage-Turner syndrome (PTS)** are conditions involving nerve damage and muscle atrophy linked to the **brachial plexus**.

Document [8], discussing HNA, explicitly states: "In **brachial plexus degeneration**, atrophy may occur in the **deltoid muscles**." Similarly, Document [11] notes that while the deltoid is often spared in Facioscapulohumeral muscular dystrophy, this sparing is not seen in other conditions affecting scapular muscles like **HNA**, implying deltoid involvement. Regarding PTS, Document [6] and [109] confirm that the condition can involve the axillary nerve, which innervates the **deltoid**, leading to weakness and atrophy. The **deltoid muscle** is anatomically described as a thick, triangular muscle (named after the Greek letter delta).

Therefore the answer is Deltoid muscle.

**Rationale**

**Short Answer**

Infraspinatus muscle

**Long Answer**

Based on the retrieved documents, **Parsonage-Turner syndrome** (PTS) involves neuropathy of the **suprascapular nerve** in 97% of cases, which innervates the **supraspinatus** and **infraspinatus** muscles. **Hereditary neuralgic amyotrophy** (HNA) is characterized to be similar to PTS in prognosis and also involves damage to the nerves of the brachial plexus.

Among the muscles affected, the **infraspinatus muscle** is explicitly described in the text as a "**thick triangular muscle**" (Document [35]). While the deltoid is also triangular and can be affected (especially in HNA or variable PTS cases), the infraspinatus is part of the muscle group (suprascapular nerve innervation) most consistently involved in PTS (97% of cases), and the specific descriptor matches the text for the infraspinatus.

Therefore the answer is Infraspinatus muscle.

**Web Search**

**Short Answer**

Infraspinatus

**Long Answer**

Based on the search results, **Parsonage-Turner syndrome** (PTS) and **hereditary neuralgic amyotrophy** (HNA) are clinically similar conditions involving inflammation and degeneration of the brachial plexus. The nerves most frequently affected are the **suprascapular nerve**, the **long thoracic nerve**, and the **axillary nerve**.

While the **deltoid** is classically described as a "thick triangular muscle" (named after the Greek letter Delta), the search results indicate that the **suprascapular nerve** (innervating the **supraspinatus** and **infraspinatus** muscles) is the **most frequently** involved nerve, affecting up to **97%** of cases (compared to ~50% for the axillary nerve/deltoid). Specific anatomy texts retrieved in the search (e.g., *Access To Surgery 500 SBA Anatomy*) explicitly describe the **infraspinatus** as a "**thick triangular muscle**". Therefore, given the higher frequency of involvement compared to the deltoid, the infraspinatus is the most likely thick triangular muscle to be affected.

Therefore the answer is Infraspinatus.

**Decision Maker**

**Short Answer**

Deltoid muscle

**Long Answer**

\## 1) Key decision criteria

1. **Anatomical correctness of the “thick triangular muscle” descriptor** (which muscle is classically described that way in standard anatomy).

2. **Clinical plausibility for *both* Parsonage-Turner syndrome (PTS) and hereditary neuralgic amyotrophy (HNA)** (i.e., a muscle commonly affected given typical brachial plexus/branch involvement).

3. **Relevance to the question prompt** (it asks for the muscle most likely affected by both conditions, not the single most commonly affected nerve in one condition).

4. **Logical consistency and evidentiary support** (no overreaching claims; avoids dubious statistics; matches the question’s framing).

---

\## 2) Step-by-step comparison of the candidate answers

\### Candidate A (Short: Infraspinatus)

- **Criterion 1 (descriptor match):**

The infraspinatus *can* be described as roughly triangular, but the phrase “thick triangular muscle” is **most classically and universally used for the deltoid** in anatomy teaching (deltoid = “triangular” shoulder cap, named after the Greek letter delta). So the descriptor fit is **weaker** than for deltoid.

- **Criterion 2 (affected by both PTS and HNA):**

Infraspinatus can be affected via **suprascapular nerve** involvement, which can occur in PTS and can occur in HNA. So it’s **plausible**, but not uniquely identifying.

- **Criterion 3 (question relevance):**

The answer leans heavily on the idea that suprascapular nerve is “most frequently involved” and therefore picks infraspinatus. But the question is asking which *thick triangular muscle* is most likely affected by **both** conditions—this points more toward a classic anatomy identification than toward a frequency argument about one nerve distribution.

- **Criterion 4 (support/accuracy):**

The “97%” claim is a red flag: PTS has variable patterns; while suprascapular involvement is common, presenting it as ~97% is not a broadly accepted, robust generalization. This weakens reliability.

**Overall:** Some plausibility, but weaker match to the “thick triangular muscle” clue and relies on a questionable frequency statistic.

---

\### Candidate B (Short: Infraspinatus muscle)

- **Criterion 1 (descriptor match):**

Same issue as Candidate A: infraspinatus can be triangular, but **deltoid is the canonical “thick triangular muscle.”**

- **Criterion 2 (affected by both PTS and HNA):**

Again plausible via suprascapular neuropathy, but not the most straightforward “both conditions” muscle.

- **Criterion 3 (question relevance):**

This candidate also prioritizes suprascapular nerve frequency in PTS rather than the classic anatomical cue embedded in the question.

- **Criterion 4 (support/accuracy):**

Repeats the “97%” claim; even if sourced somewhere, it’s not the most dependable general teaching point and risks being misleading.

**Overall:** Slightly cleaner than A, but still likely not what the question is testing and still rests on a shaky prevalence claim.

---

\### Candidate C (Short: Deltoid muscle)

- **Criterion 1 (descriptor match):**

**Excellent.** The deltoid is *the* muscle most commonly described as a **thick, triangular** muscle of the shoulder.

- **Criterion 2 (affected by both PTS and HNA):**

**Strong.** Both PTS and HNA can involve the brachial plexus and its branches; **axillary nerve** involvement is a known pattern, and axillary neuropathy directly affects the **deltoid**, producing weakness and atrophy.

- **Criterion 3 (question relevance):**

**Directly answers the question** as posed: a thick triangular muscle likely affected in brachial plexus–related neuropathies.

- **Criterion 4 (support/accuracy):**

The reasoning is anatomically and clinically coherent without hinging on a single dubious statistic. It uses a standard anatomical definition (deltoid = thick triangular) and a standard clinical pathway (brachial plexus/axillary nerve involvement → deltoid atrophy).

**Overall:** Best aligns with the anatomy clue and the clinical overlap between PTS and HNA.

---

\## 3) Are any candidates appropriate and accurate enough?

Yes—**Candidate C** is appropriate and accurate enough to select.

- It best matches the **explicit anatomical descriptor** (“thick triangular muscle”).

- It is highly plausible clinically for **both** PTS and HNA via **axillary nerve** involvement.

- It avoids overreliance on questionable prevalence figures and stays aligned with the question’s framing.

Candidates A and B are not the best choices primarily because they **fight the question’s strongest cue** (the classic “thick triangular muscle” identification) and lean on an arguably unreliable “97%” involvement claim.

---

\## 4) Final decision (selected long answer)

**Deltoid muscle**

Parsonage-Turner syndrome and hereditary neuralgic amyotrophy both involve brachial plexus–related neuropathy that can produce focal weakness and atrophy in muscles supplied by affected branches. A classic “thick triangular muscle” of the shoulder is the **deltoid muscle**, and it is commonly affected when the **axillary nerve** (a branch associated with brachial plexus pathology) is involved. Because both conditions can involve this distribution, the **deltoid muscle** is the most likely thick triangular muscle to be affected by both.

Therefore the answer is **Deltoid muscle**.

**Case of the Decision Maker Generating Direct Answers**

**Question**

What is the common name of the disease that, along with thin basement membrane disease, is a frequent cause of hematuria without other symptoms?

**Query2Doc**

**Short Answer**

IgA nephropathy

**Long Answer**

Based on the retrieved documents, **thin basement membrane disease** (also known as benign familial hematuria) is, along with **IgA nephropathy** (also known as Berger's disease), the most common cause of hematuria (specifically isolated microscopic hematuria) without other symptoms such as proteinuria or reduced kidney function.

Therefore the answer is IgA nephropathy.

**Rationale**

**Short Answer**

IgA nephropathy

**Long Answer**

Based on the provided documents, **Thin basement membrane disease** is, along with **IgA nephropathy** (also known as **Berger's disease**), the most common cause of hematuria without other symptoms (specifically isolated glomerular hematuria).

Therefore the answer is IgA nephropathy.

**Web Search**

**Short Answer**

IgA nephropathy

**Long Answer**

Based on the search results, **thin basement membrane disease** (also known as benign familial hematuria) is frequently cited alongside **IgA nephropathy** (or Immunoglobulin A nephropathy) as the most common causes of **hematuria** (blood in the urine) without other symptoms (asymptomatic hematuria). While Alport syndrome is also a cause, it is typically associated with other symptoms like hearing loss or vision impairment and a family history of kidney failure, whereas IgA nephropathy and thin basement membrane disease often present as isolated microscopic hematuria.

Therefore the answer is IgA nephropathy.

**Decision Maker**

Short Answer

Berger's disease

Long Answer

\## 1) Key decision criteria

To choose the best answer, the most important criteria here are:

1. **Directly answers what the question asks (relevance)**

- The question asks for the **common name** of the disease.

2. **Accuracy (medical correctness)**

- The disease frequently paired with thin basement membrane disease as a cause of isolated/asymptomatic hematuria is **IgA nephropathy**, whose common name is **Berger's disease**.

3. **Terminology match / precision**

- If the question asks for a *common name*, replying with only the *formal/official name* is not fully responsive.

4. **Completeness without adding incorrect claims**

- The answer should identify the disease and provide the common name clearly, without drifting into less relevant differentials.

---

\## 2) Step-by-step comparison of candidates

\### Candidate A

- **Relevance to “common name”**: Weak

- It answers **IgA nephropathy** (formal name) but does **not** provide the **common name**.

- **Accuracy**: Mostly accurate

- It correctly pairs IgA nephropathy with thin basement membrane disease as common causes of isolated hematuria.

- **Completeness/clarity**: Moderate

- Adds extra discussion (e.g., Alport syndrome) that is not required and still doesn’t give the common name.

- **Main issue**: Does not comply with the “common name” requirement.

\### Candidate B

- **Relevance to “common name”**: Better (but still not correct in the required field)

- The long answer explicitly states IgA nephropathy is “also known as **Berger's disease**,” which *does* address the common name concept.

- However, the **short answer** is still **IgA nephropathy**, not the common name.

- **Accuracy**: Accurate

- **Completeness/clarity**: Good

- Concise and correctly identifies the pairing and gives the synonym.

- **Main issue**: The question asks *for the common name*; the candidate’s actual selected answer remains the formal name.

\### Candidate C

- **Relevance to “common name”**: Similar to B (partial)

- It also mentions “also known as Berger's disease” in the long answer.

- But again, the **short answer** is **IgA nephropathy**, not the common name requested.

- **Accuracy**: Accurate

- **Completeness/clarity**: Good

- **Main issue**: Same as B—doesn’t actually provide the common name as the answer.

---

\## 3) Are any candidates appropriate and accurate enough to be selected?

**None of the three candidates is fully appropriate** because the question explicitly asks for the **common name**, and all three candidates ultimately give **IgA nephropathy** as the answer instead of the common name **Berger's disease**.

Candidates **B** and **C** come closest because they at least *mention* Berger's disease, but they still do not *answer* with it.

So I will provide my own short and long answers that directly address the question.

---

\## 4) Final decision (my generated answer)

- **Short Answer:** Berger's disease

- **Long Answer:**

The disease that is frequently listed **along with thin basement membrane disease** as a common cause of **hematuria without other symptoms (isolated/asymptomatic hematuria)** is **IgA nephropathy**, whose **common name is Berger's disease**.

Therefore the answer is Berger's disease.

## **Appendix D**

**Qualitative Comparison: Standalone Retrieval versus Full Pipeline**

This appendix provides the detailed responses underlying the qualitative analysis presented in the Analysis section. In both cases, the standalone output is the Query2Doc step using Gemini 3 Pro, and the full-pipeline output is produced by the GPT-5.2 Decision Maker, which compares the three candidate answers and selects the most suitable one. The two cases were selected to illustrate, respectively, 1) selection that improves long-answer quality without changing the short answer, and 2) selection that corrects the short answer itself.

**Case 1: Equal Short Answer, Selection of the Better-Supported Candidate**

**Question**

Are Absent tibia-polydactyly-arachnoid cyst syndrome and Charcot-Marie-Tooth disease caused by the same chromosomal abnormalities?

**Standalone**

**(Query2Doc w/ Gemini 3 Pro)**

**Short Answer (Correct)**

No

**Long Answer**

Based on the provided documents, **Absent tibia-polydactyly-arachnoid cyst syndrome** (also known as Holmes-Collins syndrome) is described as a rare hereditary disorder characterized by skeletal and neurological malformations such as missing tibia, polydactyly, and arachnoid cysts [1]. **Charcot-Marie-Tooth disease** is described as a heterogeneous group of inherited neuropathies caused by mutations in specific genes (e.g., PMP22 duplication on chromosome 17, MFN2, GJB1) affecting the peripheral nervous system [7, 24, 51, 97]. There is no information in the documents indicating they share the same chromosomal abnormalities; they are distinct conditions with different clinical presentations and underlying genetic mechanisms described.

Therefore the answer is No.

**Full Pipeline
(Query2Doc/Rationale/Web search w/ Gemini 3 Pro, Decision-making w/ GPT-5.2)**

**Short Answer (Correct)**

No

**Long Answer**

\## 1. Key decision criteria

To choose the best candidate answer, the most important criteria here are:

1. **Accuracy (genetic facts)**

- Whether each condition is correctly characterized genetically (known vs unknown chromosomal abnormality; typical inheritance; key loci for Charcot-Marie-Tooth disease).

2. **Relevance to the specific question**

- The question asks whether they are caused by the **same chromosomal abnormalities**, so the answer must directly compare the chromosomal/genetic causes.

3. **Logical consistency**

- The conclusion (“Yes” or “No”) must follow from the stated evidence.

4. **Completeness / appropriate nuance**

- Charcot-Marie-Tooth disease is genetically heterogeneous; a good answer should not imply one single cause for all cases.

5. **Avoidance of unsupported claims**

- Especially important for the rare Absent tibia-polydactyly-arachnoid cyst syndrome, where the genetic basis may be unclear.

---

\## 2. Step-by-step comparison of candidates

\### Candidate A

- **Accuracy:**

- Correctly states that **Charcot-Marie-Tooth disease** commonly involves a duplication on **Chromosome 17 (17p11.2) affecting *PMP22***, and that other chromosomes/genes can be involved.

- For **Absent tibia-polydactyly-arachnoid cyst syndrome**, it says the cause is **unknown** and “suspected autosomal recessive,” which is plausible for an ultra-rare syndrome, but it is phrased a bit broadly (“no specific gene mutations or chromosomal abnormalities have been pinpointed to date”) without the specific support details Candidate B provides.

- **Relevance:** Directly addresses “same chromosomal abnormalities” by contrasting “unknown” vs “known (e.g., 17p11.2 duplication).”

- **Nuance:** Reasonable, but slightly less careful in how it frames what has/has not been excluded for the rare syndrome.

- **Weakness:** Less specific about what investigations have been done for the rare syndrome; could be read as overly definitive (“no … have been pinpointed to date”) depending on literature updates.

\### Candidate B

- **Accuracy:**

- Correctly describes **Absent tibia-polydactyly-arachnoid cyst syndrome** as rare and **autosomal recessive**, and importantly notes that **no specific chromosomal abnormality like CMT duplications is identified** in the described context.

- Adds a helpful detail: researchers **excluded abnormalities in certain HOX genes** (this supports the claim that a particular suspected genetic mechanism was investigated and not found).

- Correctly describes **Charcot-Marie-Tooth disease** as heterogeneous and gives representative genes (*PMP22*, *MPZ*, *GJB1*, *MFN2*) and the classic **duplication of 17p11.2** for CMT1A.

- **Relevance:** Very directly compares whether there is evidence of the *same* chromosomal abnormality (there isn’t).

- **Logical consistency:** The “No” conclusion is well supported by the comparison.

- **Completeness / nuance:** Best among the three: it emphasizes heterogeneity in CMT and does not oversimplify.

- **Weakness:** Minor—“based on retrieved documents” style phrasing, but substantively it answers the question well.

\### Candidate C

- **Accuracy:** Generally correct: distinguishes the syndromes and notes CMT genetic heterogeneity with examples.

- **Relevance:** Addresses the question, but mostly by saying “no information indicates they share” rather than positively explaining the mismatch in known causes.

- **Completeness / nuance:** Adequate but the most generic of the three; adds less concrete comparative substance than Candidate B.

- **Weakness:** More vague; relies on “no information in the documents” rather than making the clearer genetic contrast.

---

\## 3. Appropriateness: is any candidate accurate enough to select?

Yes. All three conclude **No**, and that is consistent with current mainstream genetic understanding: **Charcot-Marie-Tooth disease** has well-characterized genetic causes (including chromosomal duplications such as **17p11.2** in many cases), while **Absent tibia-polydactyly-arachnoid cyst syndrome** is not known to be caused by that same chromosomal abnormality.

Among the candidates, **Candidate B** is the most appropriate because it:

- Provides the clearest, best-supported comparison,

- Properly emphasizes CMT heterogeneity,

- Avoids overclaiming by stating that no such chromosomal abnormality is identified for the rare syndrome in the provided context, and

- Includes a specific investigative detail (HOX genes excluded) that strengthens the justification.

---

\## 4. Final decision (selected best answer)

**Short Answer:**

No

**Long Answer:**

Based on the retrieved documents, **Absent tibia-polydactyly-arachnoid cyst syndrome** (also known as Holmes-Collins syndrome) is a rare **autosomal recessive** disorder described as a distinct syndrome involving limb and brain malformations, with no specific chromosomal abnormality (such as the duplications seen in CMT) identified in the text—researchers specifically excluded abnormalities in certain HOX genes. In contrast, **Charcot-Marie-Tooth disease** is a heterogeneous group of peripheral neuropathies often caused by specific genetic mutations (e.g., in *PMP22*, *MPZ*, *GJB1*, *MFN2*) or chromosomal abnormalities such as a **duplication of 17p11.2** (which causes CMT Type 1A). There is no evidence in the provided context that they are caused by the same chromosomal abnormalities.

Therefore the answer is No.

Both the standalone output and the selected candidate are scored identically under EM, yet the selected answer is more diagnostically informative in three respects. First, it supplies the precise inheritance mode and the specific chromosomal locus (17p11.2 → CMT1A) on which the question turns, rather than a generic statement of difference. Second, it surfaces the negative evidence—the explicit exclusion of HOX-gene involvement—that constitutes the actual basis for the "No" verdict, making the answer traceable to its supporting documents. Third, the Decision Maker's selection is accompanied by an explicit comparison stating why the chosen candidate is preferred over the more generic alternatives. This case corresponds to the EM-parity configuration discussed in the main text: the pipeline's advantage lies entirely in reasoning depth and explainability, neither of which is captured by EM.

**Case 2: Rectified Short Answer via Candidate Adjudication**

**Question**

What connective tissue-supporting network is affected in individuals with a mutation in the PEPD gene?

**Standalone**

**(Query2Doc w/ Gemini 3 Pro)**

**Short Answer (Wrong)**

Collagen

**Long Answer**

Based on the retrieved documents, the **PEPD** gene encodes the enzyme **prolidase** (also known as Xaa-Pro dipeptidase or peptidase D). Prolidase plays a critical role in **collagen** metabolism by recycling proline and hydroxyproline from dipeptides (breakdown products of collagen) for the resynthesis of new collagen. Collagen is described as the most abundant protein in the body, forming the rigid **support structure** or **network** essential for maintaining the integrity of **connective tissues**, skin, capillaries, and lymphatic tissue. A mutation in the *PEPD* gene leads to prolidase deficiency, which impairs this recycling process, causing the deterioration of the **collagen** network (or the collagen-rich extracellular matrix).

Therefore the answer is Collagen.

**Full Pipeline
(Query2Doc/Rationale/Web search w/ Gemini 3 Pro, Decision-making w/ GPT-5.2)**

**Short Answer (Correct)**

Extracellular matrix

**Long Answer**

\## 1) Key decision criteria

1. **Relevance to the exact wording of the question**

- The question asks: *“What connective tissue-supporting network is affected…?”*

- That phrasing strongly points to a *network outside cells that supports connective tissue*, i.e., the **extracellular matrix (ECM)**.

2. **Biological/medical accuracy about PEPD mutations**

- **PEPD** encodes **prolidase**; mutations cause **prolidase deficiency**, which disrupts **collagen turnover** and therefore impacts the **collagen-rich extracellular matrix**.

3. **Specificity and appropriateness of the entity named**

- The best answer should name the correct “network” (broad structural scaffold) rather than only a single component, unless the question clearly asks for the component.

4. **Logical consistency and completeness**

- The explanation should connect PEPD → prolidase → collagen/proline recycling → ECM/connective-tissue support.

---

\## 2) Step-by-step comparison of the candidates

\### Candidate A

- **Match to question wording (network supporting connective tissue):** Strong. It explicitly identifies the **extracellular matrix** as the supportive network.

- **Accuracy:** Correct that PEPD encodes prolidase and that prolidase deficiency disrupts collagen recycling and ECM remodeling.

- **Specificity:** Appropriately names the broader **extracellular matrix** network and notes collagen as a key component (good nuance).

- **Completeness/clarity:** Provides a coherent causal chain from gene defect to ECM disruption.

**Assessment:** Accurate and best aligned with the phrasing “connective tissue-supporting network.”

---

\### Candidate B

- **Match to question wording:** Also strong—answers **extracellular matrix**.

- **Accuracy:** Generally correct and similar to A.

- **Specificity:** Appropriate (ECM).

- **Completeness/clarity:** Adequate, but relies on a reference to “Document [13]” that is not provided here, making it slightly less self-contained than A.

**Assessment:** Also good, but slightly weaker presentation/support compared with A.

---

\### Candidate C

- **Match to question wording:** Weaker. It answers **collagen**, which is a major *component* of the network, but the question asks for the *network* that supports connective tissue (more naturally the **extracellular matrix**).

- **Accuracy:** The biology about collagen metabolism and prolidase is correct.

- **Specificity:** Somewhat too narrow for the question as written. Collagen alone is not typically referred to as the entire “connective tissue-supporting network” in the same way the ECM is.

- **Completeness/clarity:** Reasonable, and it does acknowledge the “collagen-rich extracellular matrix,” but the short answer still selects “Collagen,” which is less aligned with the prompt.

**Assessment:** Biologically related but not the best match to the asked entity (“network”).

---

\## 3) Is any candidate appropriate and accurate enough?

Yes. **Candidate A** is appropriate and accurate enough to select. It best matches the question’s phrasing (“connective tissue-supporting network”) and correctly ties PEPD/prolidase deficiency to disruption of the **extracellular matrix**.

Candidate B is similar but less self-contained. Candidate C is plausible mechanistically but answers a narrower component rather than the network requested.

---

\## 4) Final decision (selected long answer)

**Selected Answer: Candidate A**

- **Short Answer:** Extracellular matrix

- **Long Answer:** Based on the search results, the **PEPD** gene encodes the enzyme **prolidase** (also known as **peptidase D**). This enzyme is essential for the hydrolysis of imidodipeptides containing C-terminal **proline** or **hydroxyproline**, a process that is the rate-limiting step in the recycling of **collagen**.

Collagen is the most abundant structural protein in the body and a primary component of the **extracellular matrix** (ECM). Search results (specifically from MedlinePlus) describe the **extracellular matrix** as the ""lattice of proteins and other molecules outside the cell"" that ""strengthens and **supports connective tissues**."" Mutations in the *PEPD* gene lead to **Prolidase Deficiency**, which impairs the recycling of proline for new collagen synthesis and disrupts the remodeling of the **extracellular matrix**. Consequently, the specific network affected is the **extracellular matrix** (often specifically its **collagen** component).

Therefore the answer is Extracellular matrix.

In this sample, the standalone model yields an incorrect response, whereas the full pipeline successfully arrives at the correct answer. Notably, although the candidate pool included the same flawed response generated by the standalone baseline, the Decision Maker successfully filtered it out. Specifically, while the standalone baseline selected a biologically related but insufficiently precise entity (collagen, a component), the Decision Maker recognized that the question's wording ("connective tissue-supporting network") calls for the matrix as a whole. The explicit criterion driving this selection—matching the named entity to the granularity the question requests, rather than defaulting to the most salient component—is what drives this correction. Consequently, this case demonstrates that candidate adjudication can improve quantitative correctness, not merely explanation quality.

**Summary**

The two cases delineate the complementary effects of the decision-making stage, both arising from the same mechanism of criteria-based comparison among candidates. When the candidates already agree on the correct short answer, the stage promotes the most precise and best-supported one, contributing depth and explainability that EM cannot register (Case 1). When the candidates differ in the precision or granularity of the named entity, the stage's adjudication selects the better-matched answer and thereby improves EM directly (Case 2). In both regimes the value added is attributable to explicit, criteria-based selection among candidate answers.

## **Appendix E**

**Qualitative Analysis of the Direct-Generation Fallback Mechanism**

**In this section, we present a concrete exemplar and a detailed qualitative analysis illustrating how the Decision Maker responds when it adjudicates that all provided candidate answers are fundamentally incorrect. This case demonstrates the operational dynamics of the direct-generation fallback mechanism in correcting collective failure modes.**

**Question**

Are the criteria for eosinophil counts in the diagnosis of hypereosinophilic syndrome (HES) and chronic eosinophilic leukemia (CEL) identical?

**Query2Doc**

**Short Answer (Wrong)**

Yes

**Long Answer**

Based on the retrieved documents, the diagnosis of **hypereosinophilic syndrome (HES)** requires a peripheral blood eosinophil count greater than **1.5 × 10⁹/L** (or 1,500/μL) (Documents 5, 9, 19). Similarly, the diagnosis of **Chronic eosinophilic leukemia, not otherwise specified (CEL, NOS)** requires an eosinophil blood count greater than **1.5 × 10⁹/L** (or 1,500/μL) (Document 3). Both conditions share the definition of ""hypereosinophilia"" as the baseline count criterion, with the distinction lying in the presence of clonality or blast cells for CEL versus the idiopathic nature and tissue damage for HES (Documents 1, 3). While HES historically included a duration criterion (e.g., 6 months), the absolute count threshold used to define the state of hypereosinophilia in both disorders is the same.

Therefore the answer is Yes.

**Rationale**

**Short Answer (Wrong)**

Yes

**Long Answer**

Based on the retrieved documents, both **Hypereosinophilic syndrome** (HES) and **Chronic eosinophilic leukemia** (specifically CEL, NOS) are characterized by **hypereosinophilia**, which is defined as a peripheral blood **eosinophil count** greater than **1,500/μL** (or 1.5 × 10⁹/L) [1, 3, 5, 9, 19]. While the diagnostic criteria differ regarding the presence of blast cells, clonality, and tissue damage (CEL requires blasts or clonality; HES requires tissue damage and exclusion of other causes) [1, 3, 5], the criterion for the **absolute eosinophil count** threshold (>1,500/μL) is the same for both conditions.

Therefore the answer is Yes.

**Web Search**

**Short Answer (Wrong)**

Yes

**Long Answer**

Based on the **WHO 2022** classification, the criteria for **eosinophil counts** in the diagnosis of **Hypereosinophilic syndrome** (HES) and **Chronic eosinophilic leukemia** (CEL) are effectively **identical**. Both conditions require a peripheral blood eosinophil count of **??.5 ? 10??L** (1,500/?L). While HES historically required this count to persist for at least **6 months**, the WHO 2022 update reduced this duration requirement to **4 weeks** (identical to the interval often used for CEL) to facilitate earlier diagnosis and treatment, provided other exclusionary criteria (like clonality for CEL vs. idiopathic nature for HES) are met.

Therefore the answer is Yes.

**Decision Maker**

**Short Answer (Correct)**

No

**Long Answer**

\## 1) Key decision criteria

To choose the best answer, the important criteria are:

1. **Accuracy (medical correctness)**

- Correct absolute eosinophil count threshold(s) used in definitions/criteria.

- Correct handling of *persistence/duration* requirements.

- Correct recognition that **Hypereosinophilic syndrome** criteria can involve **tissue hypereosinophilia** (not only blood counts) in some modern definitions.

2. **Relevance to the exact question**

- The question asks whether the **eosinophil-count criteria** used to diagnose **Hypereosinophilic syndrome** and **Chronic eosinophilic leukemia** are **identical**.

- A fully correct answer should clarify whether “identical” holds strictly, or only in the narrow sense of the numeric threshold.

3. **Completeness and precision**

- Distinguish between:

- the **numeric threshold** used to define hypereosinophilia (commonly 1.5 × 10⁹/L = 1,500/μL), and

- the **way counts are applied** (persistence, repeated measures, allowance for tissue-only disease).

4. **Clarity and internal consistency**

- No garbled units; no unsupported claims (e.g., specific WHO 2022 timing rules stated as fact without nuance).

---

\## 2) Step-by-step comparison of candidates

\### Candidate A

- **Strengths**

- Tries to address a possible difference in *duration/persistence* criteria historically used for **Hypereosinophilic syndrome**.

- **Problems (accuracy + clarity)**

- The eosinophil count is **garbled** (“??.5 ? 10??L”), which is a major clarity/quality issue.

- It claims WHO 2022 made a **4-week duration requirement** “identical” to CEL’s interval. In reality:

- Modern approaches to **Hypereosinophilic syndrome** often use **≥1 month / repeated measurements** *or* allow diagnosis sooner when organ damage is present, and may allow **tissue hypereosinophilia** even if blood AEC is lower.

- **Chronic eosinophilic leukemia, not otherwise specified** is a myeloid neoplasm whose diagnosis does **not** hinge on an arbitrary time threshold in the same way; it hinges on **clonality/increased blasts** and exclusion of other entities.

- Overall, it **overstates “identical”** and contains formatting errors.

**Verdict:** Not reliable enough to select.

---

\### Candidate B

- **Strengths**

- Correctly states the common **numeric threshold** for hypereosinophilia: **> 1,500/μL (1.5 × 10⁹/L)**.

- Correctly notes that other diagnostic features differ (blast cells/clonality vs tissue damage/exclusion of causes).

- **Problems (precision/completeness relative to “identical”)**

- It concludes the eosinophil count criterion is “the same for both conditions,” which is **only partly true**:

- **Hypereosinophilic syndrome** diagnostic frameworks can allow **tissue hypereosinophilia** (organ infiltration) as part of criteria, and some modern formulations do **not** require a prolonged persistent blood AEC elevation if there is compatible organ disease and other causes are excluded.

- **Chronic eosinophilic leukemia, not otherwise specified** is typically framed around **persistent eosinophilia** in blood, but the diagnosis is fundamentally a **clonal myeloid neoplasm**; the “count criterion” is not applied identically in the same way as **Hypereosinophilic syndrome** frameworks that incorporate tissue-only options and/or specific repetition timing.

- So it is a bit **too absolute** in saying “identical.”

**Verdict:** Better than A, but still not strictly correct for the word “identical.”

---

\### Candidate C

- **Strengths**

- Like B, it correctly states the common numeric threshold (**> 1.5 × 10⁹/L / 1,500/μL**).

- It is more specific in naming **Chronic eosinophilic leukemia, not otherwise specified (CEL, NOS)**.

- It mentions the historical duration criterion for **Hypereosinophilic syndrome**.

- **Problems (precision/completeness relative to “identical”)**

- Same core issue as B: it treats the eosinophil-count criterion as identical without clearly acknowledging that **Hypereosinophilic syndrome** criteria may incorporate **tissue hypereosinophilia and organ involvement** such that a strict “persistent AEC ≥ 1,500/μL” blood-count requirement is not always applied identically.

- Also, **Chronic eosinophilic leukemia, not otherwise specified** does not typically use the same kind of “duration” rule as a defining feature; it’s more about clonality/blasts and classification exclusion.

**Verdict:** Slightly stronger than B in specificity, but still not fully correct given the question’s wording (“identical”).

---

\## 3) Are any candidates appropriate and accurate enough?

Because the question asks whether the **criteria are identical**, an answer that simply says “Yes” based only on the shared numeric threshold is **not strictly accurate**. The **numeric threshold** commonly referenced is similar, but the **application of eosinophil criteria** differs (persistence/repetition expectations and the role of tissue hypereosinophilia/organ involvement in **Hypereosinophilic syndrome**).

So **none** of the candidates is fully suitable as written.

---

\## 4) My own answers (short + long)

\### Short Answer

No

\### Long Answer

The eosinophil-related criteria for diagnosing **Hypereosinophilic syndrome** and **Chronic eosinophilic leukemia, not otherwise specified (CEL, NOS)** are **not strictly identical**, even though they commonly reference the same numeric level used to define hypereosinophilia.

1. **Shared numeric threshold (similarity)**

- Both conditions commonly use **hypereosinophilia defined as an absolute eosinophil count (AEC) around ≥ 1.5 × 10⁹/L (≥ 1,500/μL)** as a key reference point.

2. **How the eosinophil criteria are applied differs (why they are not identical)**

- **Hypereosinophilic syndrome** diagnostic frameworks typically require:

- hypereosinophilia **and** evidence of **organ/tissue involvement or damage attributable to eosinophils**, and

- exclusion of secondary causes.

Importantly, modern definitions may allow **tissue hypereosinophilia with organ manifestations** to satisfy the “hypereosinophilia” concept even if the peripheral blood AEC is not persistently ≥ 1,500/μL, and they often use **repeated measurements over weeks** rather than a rigid long duration requirement.

- **Chronic eosinophilic leukemia, not otherwise specified (CEL, NOS)** is a **clonal myeloid neoplasm** and is diagnosed based on:

- eosinophilia (often marked and persistent in blood), **plus**

- **evidence of clonality and/or increased blasts** (but <20%), and

- exclusion of other defined myeloid neoplasms (including those with specific rearrangements).

It does not rely on the same organ-damage-driven or tissue-eosinophilia options that can be part of **Hypereosinophilic syndrome** criteria, and it is not defined by the same style of “persistence interval” rule.

**Bottom line:** The **numeric AEC threshold** commonly cited is similar, but the **eosinophil-count criteria are not identical in a strict diagnostic sense** because **Hypereosinophilic syndrome** criteria can incorporate tissue disease/organ involvement and specific persistence/re-testing logic differently than **Chronic eosinophilic leukemia, not otherwise specified (CEL, NOS)**. Therefore the answer is No.

This case illustrates the specific failure mode that the direct-generation fallback is designed to address. All three candidates correctly identify the shared numeric threshold and correctly note that other diagnostic features differ between the two conditions. However, all three conflate the presence of a shared numeric reference point with identity of the eosinophil-count criterion as a whole. The criterion is not merely a number: it is a diagnostic construct that includes how and over what period counts are measured, and whether tissue-based evidence may substitute or supplement peripheral blood-count evidence. By applying explicit criteria-based adjudication—evaluating each candidate against defined standards of accuracy, precision, and relevance to the question's exact framing—the Decision Maker surfaces this collective insufficiency, correctly determines that no candidate is adequate, and produces a response that addresses the question as posed. This case therefore demonstrates that the direct-generation fallback contributes in situations where retrieval-stage consensus is misleading rather than merely incomplete or imprecise.
